# Can YouTube be used as an educational tool in lymphedema rehabilitation?

**DOI:** 10.1186/s40945-022-00130-9

**Published:** 2022-03-03

**Authors:** Okan Küçükakkaş, Buğra İnce

**Affiliations:** Department of Physical Medicine and Rehabilitation, Faculty of Medicine, Bezmialem Vakıf University, 34093 Istanbul, Turkey

**Keywords:** Internet, Patient education, Therapy, Lymphedema, Massage, Exercise

## Abstract

**Background:**

Lymphedema is defined as the abnormal accumulation of interstitial fluid and fibro-adipose tissues resulting from injury, infection, or congenital abnormalities of the lymphatic system. The gold standard approach in the treatment of lymphedema is Complete Decongestive Therapy and it has many components that require practical knowledge and skills. YouTube can be a useful tool to provide these skills to healthcare professionals and patients. The aim of this study was to examine the videos about lymphedema rehabilitation on YouTube and analyze their technical features, sources, contents, educational value and reliability.

**Methods:**

The YouTube database was searched using the “lymphedema rehabilitation”, “lymphedema treatment”, “complete decongestive therapy”, “lymphedema massage”, and “lymphedema exercises” keywords. Two reviewers (Physical medicine and rehabilitation specialist) assessed videos for educational quality using a Global Quality Scale (GQS). To evaluate the reliability the 5-point Discern scale was used.

**Results:**

A total of 90 videos, which met the inclusion criteria were included in the analysis. The mean duration of the videos was 8.9 ± 10.5 min. The mean number of daily views was 22.7 ± 47.1 for a day. The majority of the videos were created to inform patients (57.8%).The uploaders were mostly private healthcare institutions or healthcare professionals (65.6%). Information providers were lymphedema therapists mostly (63.3%). Manual lymphatic drainage was observed to stand out as the most mentioned lymphedema rehabilitation component on YouTube. The mean of reliability and GQS scores of the videos were 2.2 ± 1.0 and 2.7 ± 1.0, respectively.

**Conclusions:**

The biggest obstacle for YouTube to be an excellent source of information is that it hosts large volumes of uncontrolled and low-quality data. When Youtube content related to lymphedema rehabilitation was examined, it was observed that many videos were quite insufficient and incomplete even though there were useful videos. If careful controlling measures are implemented and if medical videos aim to meet reliability and GQS criteria, YouTube can become an effective and useful source of information for lymphedema rehabilitation.

## Background

Lymphedema is a progressive pathological condition characterized by the accumulation of protein-rich fluid in the interstitial space due to inadequate lymph drainage. It results from decreased lymphatic transport capacity and/or increased lymphatic load. Primary lymphedema occurs due to malformations, developmental retardations, or acquired disorders of the lymphatic circulatory system. Secondary lymphedema is a more common condition occurring after lymph node dissection and radiotherapy; which are applied for the treatment of several diseases such as breast cancer, urogenital system cancers, colorectal cancers, melanoma, and head and neck cancers [1]. Lymphedema is associated with aesthetic deformations, physical discomfort due to oedema, impaired/limited functioning, potential attacks of lymphangitis/cellulite, and psychological stress; affecting the quality of lives of patients unfavourably [2–5].

Lymphedema was considered an incurable disorder previously; however, several treatment options are available today [6]. International Society of Lymphology (ISL) recommends complete decongestive therapy (CDT) as the international standard of care for lymphedema treatment [6, 7]. CDT comprises several components such as manual lymphatic drainage (MLD), compression therapy, therapeutic exercises, skincare, patient training, and their combinations. The effectiveness of CDT in edema reduction has been demonstrated not only in mildly affected and early-stage patients but in more severely affected and chronic lymphedema patients as well. Therefore, CDT has taken its place as the standard of care for the treatment of lymphedema in the current guidelines [7–11].

YouTube is a popular video sharing site that is widely used worldwide, allowing users to share and watch videos. It can serve as an effective tool to obtain and disseminate health-related information as it offers a large variety of videos free of charge. YouTube can serve as a education tool for patients and health professionals; as well as being a readily accessible source of information for patients looking for information about their health problems. However; its mechanisms for controlling the content, information quality, and the correctness of the information in the posted videos are considerably limited. Therefore, the reliability of information is doubtful and there is a potential risk of disseminating misleading information [12, 13].

In an area such as lymphedema rehabilitation where practical skill is very important a video sharing environment like YouTube can be quite useful. Information can be provided to both health professionals and patients, especially in subjects such as bandaging, MLD, self-massage and exercise. For these reasons, we designed this study to analyse the reliability and educational quality of the content of YouTube videos related to lymphedema rehabilitation. In addition, we aimed to examine the content, sources and technical features of the videos and to analyze their associations with reliability and educational quality levels.

## Methods

### Selection of videos

To access the majority of videos about lymphedema rehabilitation; a search was conducted on YouTube on April 1, 2021, using the following keywords; including “lymphedema rehabilitation”, “lymphedema treatment”, “complete decongestive therapy”, “lymphedema massage”, and “lymphedema exercises” (www.youtube.com). During the keyword search, the YouTube search settings were as follows: upload date, a special time interval was not specified; type, video; duration, no limits were set; and sorting criteria, videos were listed in decreasing order of relevance. This search strategy was used in the literature by many other studies on YouTube [14–16]. Studies show that a large percentage of users review only videos listed on the first three pages of query results [17]. Therefore, 60 videos listed on the first 3 pages for each keyword were examined. Of the 300 videos reviewed; irrelevant videos, duplicate videos, videos in a language other than English, and videos with inappropriate sound recording were excluded.

### Video parameters

The length, visual quality, and the upload date of the videos, total number of views, daily number of views, and the numbers of likes and dislikes were noted to define the properties of each video. To evaluate the popularity of the videos, we use the “Video Power Index” (VPI) to assess both the view and the like ratio of the videos. The VPI was calculated as follows: first, calculate the like ratio (like*100/[like+dislike]) and the view ratio (number of views/days); then, the VPI is equal to the like ratio*view ratio/100 [18].

### Video content, used resources, and references

The videos were categorized by their content, the uploader, and the informer type. Video types were categorized by the general purpose of the video as follows: (1) Patient information videos, (2) Educational videos for healthcare professionals, and (3) Commercials and promotional videos. Video uploaders were categorized under the following titles: (1) private health institutions or a health professional, (2) university, (3) hospital, and (4) manufacturers of lymphedema rehabilitation equipment. Information providers were categorized under the following titles: (1) physician, (2) lymphedema therapist, (3) massage therapist, (4) manufacturing company representatives, and (5) unidentified. Videos were compared in terms of Global Quality Scale (GQS) and reliability scores according to these categories, and possible differences were investigated. Also, the types of lymphedema rehabilitation components (MLD, exercise etc.) were noted for each video.

### Assessment of educational quality and reliability

The educational quality of YouTube videos included in the study was evaluated with the GQS [19]. GQS is a five-point scale; where the lowest score is 1 and the highest score is 5. It was designed as a tool for the assessment of internet-based resources. This scale allows investigators to evaluate the flow, ease-of-use, and quality of videos at this scale. For a video; scores of 4 or 5 indicate high quality, 3 indicates intermediate quality, and 1 or 2 points indicate low quality [19]. The reliability of videos was evaluated with the modified DISCERN instrument; which makes the assessment at 5 levels. This tool comprises five “yes or no” questions. Each “yes” answer receives a score of 1 point and the highest score is 5 (Table 3). The DISCERN tool was developed for the evaluation of written health information [20]. This modified tool has been used in many studies especially to evaluate the reliability of YouTube content [13–17].

### Ethical declaration

This study did not include any human participants or animals. Only public videos on YouTube were evaluated for this study. Therefore, ethics committee approval was not required. Similar studies in the literature follow the same rationale, too [16–21].

### Statistical analysis

IBM SPSS Statistics 24 (IBM Corp. Armonk, NY) package program was used for the statistical analysis of the study data. Median (minimum-maximum), numbers, and percentages were used for summarizing the descriptive data. The distribution of the data was evaluated with the Shapiro-Wilk test and it was determined that the datas were not normally distributed (*p* < 0.05). Continuous variables in subgroups of contents, uploader and presenter were compared with the Kruskal-Wallis and Mann-Whitney U tests. The relationships of GQS and reliability scores with each other and with the technical parameters of the videos were assessed using Spearman’s correlation coefficient and values interpreted as follows: excellent at least 0.9, high 0.7 to 0.89, moderate 0.50 to 0.69, fair 0.26 to 0.49, and little or no relationship less than 0.25. Inter-observer agreement was assessed using the kappa coefficient. *P*-values of less than 0.05 were considered significant.

## Results

### Technical properties of the videos

A total of 90 videos, which met the inclusion criteria and which were uploaded on the dates between 19/09/2007–01/04/2021 were included in the analysis. Cohen’s kappa score for the interobserver agreement was 0.86 (0.66–1.00 CI). The duration of the videos ranged from 29 s to 58.4 min. The video with the highest number of views was watched 394,987 times. It was uploaded by a lymphedema specialist, providing information about lymphedema exercises and self-massaging. This video had the highest number of daily views; which was 353. Also, it received the highest number of likes and dislikes; which were 6652 and 223, respectively. The mean VPI score of all videos were 24.7 ± 48.1 (0.05 to 342.45).

The image quality was examined with pixel values (p) in the range from 240p to 1080p. The pixel value is the smallest controllable unit composing digital images [22]. The average image quality of the videos was calculated as 793.5p. The technical characteristics of the videos are presented as means of measured parameter values in Table 1.

**Table 1:** Technical characteristics of videos

	Minimum	Maximum	Mean ± SD	Median	Percentiles		
					25	50	75
**Duration (minute)**	0.48	58.4	8.9 ± 10.5	4.8	2,4	4.8	10.5
**Image quality (pixel)**	240	1080	797,3 ± 270.0	720.0	720.0	720.0	1080.0
**Number of total views**	9	394,987	35,165.6 ± 65,553.8	8108.0	818.2	8108.0	31,366.7
**Number of daily views**	0.02	353.9	22.7 ± 47.1	4.7	0.4	4.7	24.3
**Likes**	0	6652	268.8 ± 781.9	30.5	3.0	30.5	187.0
**Dislikes**	0	223	14.2 ± 32.4	2.0	0.0	2.0	10.0
**Video Power Index**	0.05	342.45	24.7 ± 48.1	5.8	0.8	5.8	28.7

Regarding the video types, the majority of videos were developed to provide information for patients (57.8%). These videos were followed by educational videos for healthcare professionals (26.7%) and video commercials (15.6%). The uploaders were mostly private healthcare institutions and / or healthcare professionals (65.6%). Information providers were lymphedema therapists mostly (63.3%). Details about the video contents are presented in Table 2.

**Table 2:** Detailed analysis of video contents

	n (%)	Reliability	GQS
**Video type**			
Information video for patients	52 (57.8)	2.1	2.6
Education video for the healthcare professional	24 (26.7)	3.0	3.5
Lymphedema product commercial	14 (15.6)	1.2	1.8
*p* value		***p*** < 0.001*	***p*** < 0.001*
**Uploader**			
Private healthcare institution / professional	59 (65.6)	2.1	2.6
University	16 (17.8)	2.5	3.5
Hospital	10 (11.1)	2.8	2.7
Lymphedema product manufacturer	5 (5.6)	1.2	2.0
p value		0.007*	0.004*
**Presenter**			
Lymphedema therapist	57 (63.3)	2.3	2.9
Massage therapist	13 (14.4)	1.8	2.5
Physician	6 (6.7)	3.0	2.5
Manufacturer representative	5 (5.6)	1.0	1.6
Unknown	9 (10.0)	2.3	3.1
p value		0.006*	0.023*

**Subgroup analyzes (Mann Whitney U analysis with Bonferoni correction)**

**Video type**

***Reliability and GQS scores =*** *There were significant differences between all video type subgroups* ***p < 0.001***

**Uploder**

***Reliability scores =*** *Universities-Manufacturer representative* ***p = 0.001***

***GQS scores =*** *Private healthcare providers-universities* ***p = 0.001***

**Presenter**

***Reliability and GQS scores =*** *lymphedema therapists- Manufacturer representative* ***p = 0.001***

*GQS* Global quality score

*Kruskal-Wallis one-way analysis of variance

When videos were examined for the components of lymphedema rehabilitation, it was observed that videos providing information about MLD (*n* = 50) were the most common. This most common video content was followed by lymphedema exercises (38), compression garments (36), self-massage (32), bandaging (31), skin care (24), losing weight (10) and pneumatic devices (4).

The mean of reliability and GQS scores of the videos were 2.2 ± 1.0 and 2.7 ± 1.0, respectively. The videos were grouped according to their GQS scores as a result of educational quality assessment as follows: poor quality; 7 (7.8%), generally sparse quality; 37 (41.1%), moderate quality; 21 (23.3%), good quality; 19 (21.1%) and excellent quality; 6 (6.7%). When the reliability scores were examined, the first question was provided in almost all videos, although the second and fourth questions were insufficient in the vast majority of the videos. Detailed review of the reliability scores were given in Table 3. When the reliability and GQS scores of the videos were compared for the content, uploaders, and presenters; a significant difference was observed across the groups. The highest scores of reliability and GQS were observed in the education videos for the healthcare professional. These scores were also high in the videos; which were uploaded by universities, hospitals and which were presented by lymphedema therapists or physicians. Sub-group analyses for video contents were done by Mann Whitney U analysis (with Bonferoni correction). In the video types sub-group analysis, a significant difference was observed between all groups (*p* < 0.001). In the uploader sub-group analysis, a significant difference was observed between the universities and manufacturers in reliability scores. In GQS scores, there was a significant difference between private healthcare providers and universities (*p* < 0.01). In presenter sub-group analysis, there was a significant difference between lymphedema therapists and manufacturer representative scores in reliability and GQS scores (*p* < 0.001). The detailed analyses of both of these scores are presented in Table 2.

**Table 3:** Distribution of videos in terms of meeting reliability criteria

	Reliability (1 point per question answered yes)	***n*** = 90
**1**	Is the video clear, concise, and understandable?	88
**2**	Are valid sources cited? (from valid studies, physiatrists or rheumatologists)	10
**3**	Is the information provided balanced and unbiased?	69
**4**	Are additional sources of information listed for patient reference?	12
**5**	Does the video address areas of controversy/uncertainty?	23

The relationships of these scores with each other and with the technical parameters of the videos were examined. There was a moderate correlation between reliability and GQS scores. The reliability scores were moderately correlated with the duration of the videos and they were fairly correlated with the number of likes. GQS scores were moderately correlated with the video duration, the number of dislikes and VPI scores and were fairly correlated with the number of likes, daily view rate and the video image quality. The results of the correlation analysis are presented in Table 4.

**Table 4:** Spearman correlation analysis between datas

	Reliability	GQS
**Daily view rate**	0.145	0.442**
**Likes**	0.285**	0.497**
**Dislikes**	0.250*	0.511**
**Lenght**	0.616**	0.625**
**Video quality**	0.226*	0.257*
**Reliability**	1.000	0.642**
**GQS**	0.642**	1.000
**VPI**	0.125	0.450**

*GQS* Global quality score, *VPI* Video Power Index,

Reliability; Obtained by evaluating videos with the 5 point modified DISCERN tool

* = *p* < 0.05, ** = *p* < 0.01

## Discussion

Lymphedema is a chronic and progressive condition, increasing the risk of recurrent infections and inducing changes in the fibrous tissue and keratin structure in the affected extremities. Also; it causes serious functional limitations and psychological problems in patients, reducing the quality of life and self-esteem [23–25]. Especially in developed countries, cancer surgery and treatment seems to be the most common cause of secondary lymphedema. The risk of developing lymphedema depends upon the type of surgery performed, individual patient factors such as obesity or weight gain after surgery, treatment factors such as radiation or some types of chemotherapy, and complications after surgery, including infections or fluid collections. Lymphedema is associated with growing social and economic impacts. A treatment program should be started immediately after the diagnosis regardless of the severity and stage of lymphedema [26].

The gold standard of treatment for lymphedema is CDT. An effective CDT should be applied by competent healthcare professionals knowledgeable about all stages of the therapy and it requires the participation of well-acknowledged patients and their relatives, who are aware of their responsibilities. In particular, MDL and compression bandaging therapy are applications that require special training and should be performed by trained therapists. Application of such treatments by unauthorized persons will not only fail to obtain effective results but also may lead to many complications. Treatment components; including the use of compression garments, lymphedema exercises, self-massage techniques, skincare, weight loss, and the use of pneumatic devices require the active participation of the patient and patient relatives. However, improper application of any of these treatment components can easily impair improvements achieved with MLD and bandaging. Therefore, lymphedema rehabilitation is a comprehensive process that can only advance with the participation of the physician, therapist, patient, and patients’ relatives (if necessary/or relevant) [1].

YouTube includes vast sources of information that can contribute to the education and self-development of all individuals involved in the lymphedema rehabilitation process. It can contribute to lymphedema rehabilitation with both educational videos for health professionals and information providing videos for patients. However; the sufficiency and correctness of the video content in YouTube raise some concerns as it is an unsupervised environment. Particularly, the contents of video commercials used for product advertisement may not be objective and they may mislead patients. For these reasons, we evaluated YouTube videos about lymphedema rehabilitation for their reliability and educational quality.

In our study, we analyzed 90 videos, most of which were uploaded by private healthcare institutions. The examination of the GQS scores showed that 49.1% of the videos were of either moderate or better quality and they were considered beneficial. Of these videos; six videos achieved a full 5 points in GQS score, indicating excellent quality. In the investigation of the reliability, it was observed that the majority of the videos met the 1st and 3rd criteria adequately but the remaining criteria could not be met by the majority of the videos. We observed that more than half of these videos were technically insufficient and their educational value was poor. Especially; potential risks, indications, contraindications, and the source of the provided information were not available in the content of the majority of the videos.

The examination of the video contents revealed that the reliability and GQS scores were especially high in the educational videos for health professionals. When the uploaders are examined; the videos uploaded by universities had higher reliability scores compared to the videos uploaded by manufacturers. Also, the videos uploaded by universities had higher GQS scores compared to the videos uploaded by private healthcare institutions (*p* < 0.01). In regard to the information providers in the videos, the scores of lymphedema-therapists were significantly higher compared to manufacturing company representatives (*p* < 0.01). We performed correlation analysis to identify other factors that may be related to the educational value and reliability of the videos. Video duration appeared to correlate with both scores. This relationship can be expected since short videos do not meet the factors that affect reliability and educational quality scores such as sufficient information, discussion and resources. Videos with higher reliability were associated with higher likes, as expected. GQS scores, on the other hand, were found to be particularly associated with the number of dislikes and VPI scores. This relationship is understandable because videos with high educational quality often have higher viewing rates. However, some points are unclear, such as why it is more closely related to the number of dislikes than the number of likes. Therefore, new studies are needed to better analyze the relationships of GQS and reliability scores of YouTube video contents with these types of parameters.

MLD was observed to stand out as the most mentioned lymphedema rehabilitation component on YouTube. MLD videos were followed by videos about exercises, compression garments, self-massage, bandaging, skincare, weight loss, and pneumatic devices in decreasing order of frequency. These treatment options with variable efficacy are mostly applied in combinations, taking into account the characteristics of the patient and lymphedema [6].

This study has several limitations. First, a subjective criteria was used to evaluate the videos, as there are as of yet no validated tools for assessing video data. Second, the evaluated videos were sorted by relevance, which is the YouTube default. This relevance may have been affected by advertisements, and the results may be different when sorted with another standard. Lastly, these results demonstrate the quality of information at one point in time, and results may change with time as videos are added or removed.

### Implications to the clinic

Consensus reports developed by the ISL provide guiding principles for the treatment [7]. However, each patient has different features. Therefore, treatment programs should be designed for each patient on an individual basis. The medical history, history of cancer, the stage of lymphedema, the lifestyle of patients, and patient expectations should be taken into consideration. When YouTube content related to lymphedema rehabilitation was examined, it was observed that many videos were quite insufficient and incomplete even though there were useful videos. Therefore, information sources such as YouTube should definitely be evaluated by expert health teams and patients should be guided accordingly considering these factors.

Recently, the difficulties experienced by patients in reaching health institutions, especially with the Covid-19 pandemic, have highlighted the concept of telerehabilitation. Telerehabilitation can be defined as the delivery of medical rehabilitation services at a distance using electronic information and communication technologies. Quality Youtube content can also be a good helper in telerehabilitation practices especially for lymphedema rehabilitation [27].

## Conclusion

The biggest obstacle for YouTube to be an excellent source of information is that it hosts large volumes of uncontrolled and low-quality data [14]. In fact, videos uploaded under the headings of like sexuality, violence, racism, and copyrights are subject to a specific checking process. However, this type of control is not in question especially in medical videos. If careful controlling measures are implemented and if medical videos aim to meet reliability and GQS criteria, YouTube can become an effective and useful source of information for lymphedema rehabilitation. For this reason, it is very important that professional healthcare providers create new videos about lymphedema rehabilitation and direct both colleagues and patients to these contents.

## Data Availability

Not applicable.

## References

[1] Aras M, Baday D (2016). Treatment of lymphedema: general aspects. Turkiye Klinikleri J PM&R-Special Topics.

[2] Liao S, Padera TP (2013). Lymphatic function and immune regulation in health and disease. Lymphat Res Biol.

[3] Paolucci T, Bernetti A, Bai AV, Segatori L, Monti M, Maggi G, Ippolitoni G, Tinelli L, Santilli V, Paoloni M, Agostini F, Mangone M (2021). The sequelae of mastectomy and quadrantectomy with respect to the reaching movement in breast cancer survivors: evidence for an integrated rehabilitation protocol during oncological care. Support Care Cancer.

[4] Paolucci T, Bernetti A, Paoloni M, Capobianco SV, Bai AV, Lai C, Pierro L, Rotundi M, Damiani C, Santilli V, Agostini F, Mangone M (2019). Therapeutic Alliance in a Single Versus Group Rehabilitative Setting After Breast Cancer Surgery : Psychological Profile and Performance Rehabilitation. Biores Open Access.

[5] Paolucci T, Bernetti A, Bai AV, Capobianco SV, Bonifacino A, Maggi G, Ippolitoni G, Tinelli L, Santilli V, Agostini F, Paoloni M, Mangone M (2021). The recovery of reaching movement in breast cancer survivors: two different rehabilitative protocols in comparison. Eur J Phys Rehabil Med.

[6] Executive Committee of the International Society of Lymphology (2020). The diagnosis and treatment of peripheral lymphedema: 2020 consensus document of the International Society of Lymphology. Lymphology.

[7] Committee E (2016). The diagnosis and treatment of peripheral lymphedema: 2016 consensus document of the International Society of Lymphology. Lymphology.

[8] Zuther JE, Norton S (2017). Lymphedema management: the comprehensive guide for practitioners.

[9] Lawenda BD, Mondry TE, Johnstone PA (2009). Lymphedema: a primer on the identification and management of a chronic condition in oncologic treatment. CA Cancer J Clin.

[10] Rockson SG (2008). Diagnosis and management of lymphatic vascular disease. J Am Coll Cardiol.

[11] Lasinski BB, Thrift KM, Squire D, Austin M, Smith K, Wanchai A, Greeen J, Stewart B, Cormier J, Armer J (2012). A systematic review of the evidence for complete decongestive therapy in the treatment of lymphedema from 2004 to 2011. PM&R.

[12] Lewis SP, Heath NL, Sornberger MJ, Arbuthnott AE (2012). Helpful or harmful? An examination of viewers' responses to nonsuicidal self-injury videos on YouTube. J Adolesc Health.

[13] Dubey D, Amritphale A, Sawhney A, Dubey D, Srivastav N (2014). Analysis of YouTube as a source of information for West Nile virus infection. Clin Med Res.

[14] Lee H, Choi A, Jang Y, Lee JI (2018). YouTube as a learning tool for four shoulder tests. Prim Health Care Res Dev.

[15] Smith PE, McGuire J, Falci M, Poudel D, Kaufman R, Patterson M, Pelleschi B, Shin E (2019). Analysis of YouTube as a source of information for diabetic foot care. J Am Podiatr Med Assoc.

[16] Tolu S, Yurdakul OV, Basaran B, Rezvani A (2018). English-language videos on YouTube as a source of information on self-administer subcutaneous anti-tumour necrosis factor agent injections. Rheumatol Int.

[17] Singh AG, Singh S, Singh PP (2012). YouTube for information on rheumatoid arthritis—a wakeup call?. J Rheumatol.

[18] Zhang S, Fukunaga T, Oka S, Orita H, Kaji S, Yube Y, Yamauchi S, Kohira Y, Egawa H (2020). Concerns of quality, utility, and reliability of laparoscopic gastrectomy for gastric cancer in public video sharing platform. Ann Transl Med.

[19] Bernard A, Langille M, Hughes S, Rose C, Leddin D, Van Zanten SV (2007). A systematic review of patient inflammatory bowel disease information resources on the world wide web. Am J Gastroenterol.

[20] Charnock D, Shepperd S, Needham G, Gann R (1999). DISCERN: an instrument for judging the quality of written consumer health information on treatment choices. J Epidemiol Commun Health.

[21] Kocyigit BF, Nacitarhan V, Koca TT, Berk E (2019). YouTube as a source of patient information for ankylosing spondylitis exercises. Clin Rheumatol.

[22] Blinn JF (2005). What is a pixel?. IEEE Comput Graph Appl.

[23] Smoot B, Wong J, Cooper B, Wanek L, Topp K, Byl N, Dodd M (2010). Upper extremity impairments in women with or without lymphedema following breast cancer treatment. J Cancer Surviv.

[24] McWayne J, Heiney SP (2005). Psychologic and social sequelae of secondary lymphedema: a review. Cancer.

[25] Paskett ED, Naughton MJ, McCoy TP, Case LD, Abbott JM (2007). The epidemiology of arm and hand swelling in premenopausal breast cancer survivors. Cancer Epidemiol Biomark Prev.

[26] Shih Y-CT XY, Cormier JN (2009). Incidence, treatment costs, and complications of lymphedema after breast cancer among women of working age: a 2-year follow-up study. J Clin Oncol.

[27] Boldrini P, Bernetti A, Fiore P (2020). SIMFER Executive Committee, SIMFER Committee for International Affairs. Impact of COVID-19 outbreak on rehabilitation services and Physical and Rehabilitation Medicine physicians' activities in Italy. An official document of the Italian PRM Society (SIMFER). Eur J Phys Rehabil Med.

